# Isotopically Enriched Lithium Fluoride Crystals for Detection of Neutrons with the Fluorescent Track Technique

**DOI:** 10.3390/ma17205029

**Published:** 2024-10-14

**Authors:** Małgorzata Sankowska, Paweł Bilski, Mariusz Kłosowski, Anna Kilian, Wojciech Gieszczyk, Barbara Marczewska

**Affiliations:** Institute of Nuclear Physics, Polish Academy of Sciences PAN (IFJ PAN), Radzikowskiego 152, 31-342 Krakow, Poland; malgorzata.sankowska@ifj.edu.pl (M.S.); mariusz.klosowski@ifj.edu.pl (M.K.); anna.kilian@ifj.edu.pl (A.K.);

**Keywords:** FNTD, lithium fluoride, isotopic composition, neutron detection, ionizing radiation, cosmic radiation

## Abstract

In this work, the properties of LiF crystals grown using Li of different isotopic compositions are described from the standpoint of their application as fluorescent nuclear track detectors used in measurements in the neutron radiation fields. The crystals were grown using two techniques: the Czochralski method and the micro-pulling-down method. Three isotopic compositions of Li were studied: natural, highly enriched in ^6^Li, and highly enriched in ^7^Li. It was found that ^6^LiF detectors are about six times more sensitive to thermal (low energy) neutrons than natural LiF, which significantly decreases the lower detection limit. ^7^LiF detectors are insensitive to thermal neutrons, which makes it easier to detect tracks due to other radiation modalities, such as energetic ions or nuclei recoiled in collisions with high-energy neutrons. Besides the response to neutron radiation, no other significant differences in the crystal properties were identified, irrespective of the isotopic composition and crystal growth method employed.

## 1. Introduction

**Figure 1:**
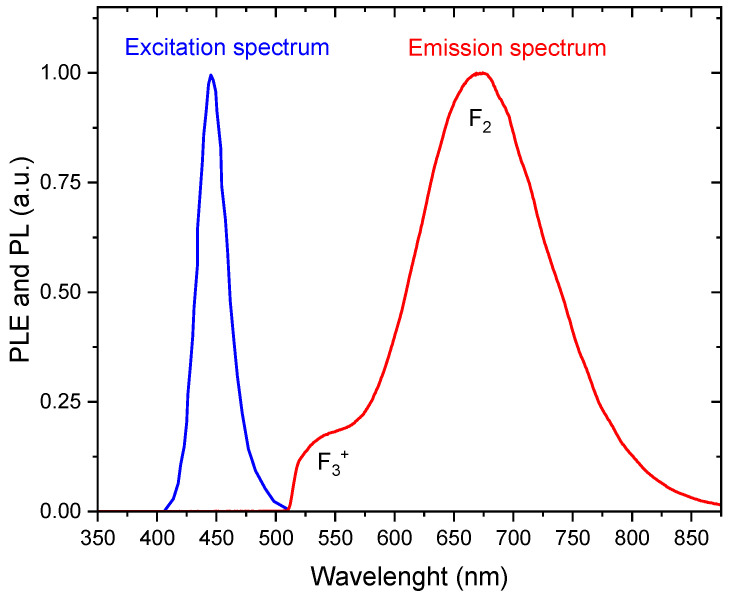
Photoluminescence excitation (PLE) and emission (PL) spectra for LiF crystal after exposure to Sr-90/Y-90 beta source. The emission spectrum was measured using a 505 nm long-pass filter to cut off the excitation light.

Fluorescent nuclear track detection (FNTD) is a radiation measurement method that has advanced over the last nearly twenty years. Originally, it was developed with aluminum oxide crystals doped with magnesium and carbon [1,2,3], which, for a long time, was the only material suitable to be used for this purpose. In recent years the FNTD method was also successfully implemented using undoped lithium fluoride crystals [4,5]. The principle of the FNTD method is based on the radiation-induced photoluminescence of the color centers created in the crystal lattice by ionizing particles along their path through a crystal. When such centers are subsequently excited by illumination with the light of a specific wavelength, they emit light of a longer wavelength. In the case of LiF, the color centers useful for this purpose are F_2_ (two anion vacancies connected with two electrons) and F_3_^+^ (three anion vacancies connected with two electrons) crystal lattice defects. The excitation wavelength is about 440 nm, and the main emission band peaked at about 670 nm (Figure 1). With the use of a fluorescent microscope at a high magnification and a very sensitive digital camera, it was possible to obtain images of tracks produced in LiF single crystals by particles ranging from protons to the heaviest nuclei [6,7] with a resolution of about 0.4 µm. The intensity of the fluorescent tracks depends on the ionization density (radiation dose deposited locally, in the submicrometric scale). An important feature is that the color centers in LiF are very stable, which allows for measurements to be performed even several years after irradiation.

In the field of radiation measurements and dosimetry, the neutrons constitute one of the most difficult tasks [8]. Neutrons, having no electric charge, do not ionize matter directly. For their detection, some nuclear reactions caused by neutrons need to be used. One of the reactions most widely exploited for this purpose is neutron capture by ^6^Li nuclei:

(1) n+Li6 → α+H3+4.78 MeV

The products of this reaction—an alpha particle and a tritium nucleus—are directly ionizing particles, which can be detected with various methods. The microscopic cross-section (a measure of the probability of a nuclear reaction) is very high for low-energy neutrons (called thermal neutrons as they remain in thermal equilibrium with a surrounding temperature), reaching a value of about 940 barns for energy of 0.025 eV, and decreases with increasing energy. Natural lithium contains about 7.5% of ^6^Li. The remainder consists of a ^7^Li isotope, which does not show a high cross-section for reactions with neutrons (see Figure S1 in Supplementary Material).

**Figure 2:**
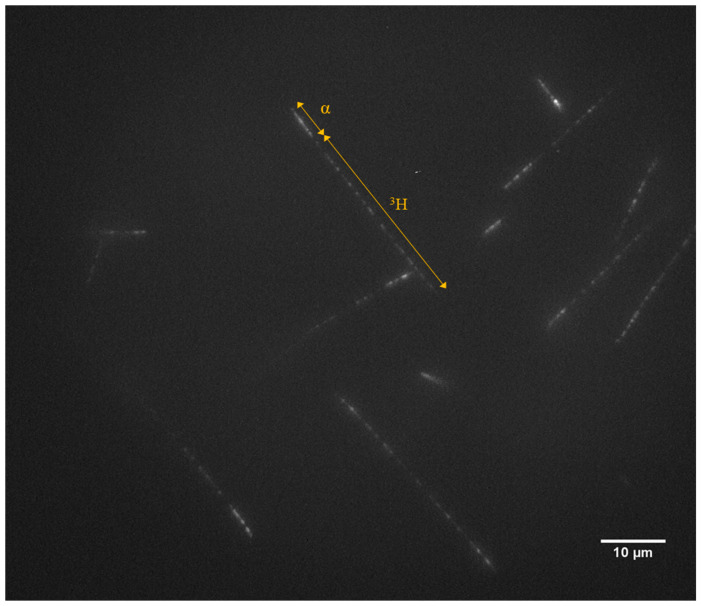
Examples of the fluorescent tracks created by the products of (n,α) reaction at ^6^Li: alpha particle and tritium nucleus in LiF crystal enriched with ^6^Li. Thermal neutron fluence around 8.6 × 10^4^ n.cm^−2^. Maximum intensity projection from images taken at depths from 13 µm below surface to 18 µm below the crystal surface (see Section 2.3 for details).

Detection of neutrons with LiF fluorescent nuclear track detectors has already been successfully attempted [9]. The products of the (n,α) reaction with ^6^Li nuclei create some characteristic in LiF, directed in opposite directions, tracks: brighter and shorter (6 µm) alpha particle and longer (33 µm) tritium nucleus (Figure 2). The LiF detectors were tested as personal dosimeters, i.e., irradiated with neutrons in a reference calibration laboratory on a phantom simulating the human body. In this way, they could measure neutrons reflected and moderated by the body (so-called albedo method [10]). The tests of such an approach revealed that using LiF detectors, it is possible to measure neutron doses at least as low as 1 mSv. However, due to the low density of tracks to achieve good statistics, a large area of a crystal needed to be scanned, which is a time-consuming process.

All these results were obtained with the crystals made from Li with natural isotopic composition. The number of created tracks is equal to the number of neutrons captured at the ^6^Li nuclei, which, in turn, depends directly on the number of ^6^Li nuclei per unit volume. Therefore, the way to increase the detection efficiency of LiF fluorescent track detectors could be by producing detectors using lithium enriched in ^6^Li. Such an approach is used in thermoluminescent dosimetry of neutrons, where polycrystalline ^6^LiF:Mg,Ti detectors are commonly applied [11]. The main goal of this work was, therefore, to grow LiF single crystals highly enriched in ^6^Li isotopes and to investigate their properties as fluorescent track detectors of neutrons.

In some applications, the tracks created in LiF by the products of the (n,α) reaction at ^6^Li nuclei may be a disturbing factor. This is, e.g., the case of measurements of cosmic radiation in space. The cosmic radiation spectrum consists of a variety of high-energy ions, and LiF track detectors have already been used for measurements in several space missions. The analysis of fluorescent tracks from space exposures was, however, found to be somewhat hampered by the presence of a number of neutron-induced tracks superimposed on those of ions. Another example is the detection of high-energy neutrons. Such neutrons may produce tracks in LiF by colliding with Li and F nuclei, which are sufficiently light to acquire a significant amount of kinetic energy in such collisions. The energy of a recoil nucleus after a collision with a neutron is described by the equation:

(2) $$E_{r}=\frac{4A}{{\left(A+1\right)}^{2}}\;E_{n}{cos}^{2}\theta$$

where

A—mass number of an isotope,
$E_{n}$—neutron energy,
θ—scattering angle.

The maximum recoil energy (for θ=0) is therefore equal to

(3) EmaxLi7=0.44En

(4) EmaxF19=0.19En

This means that, for example, a neutron with an energy of 5 MeV may impart even an energy of 2.2 MeV to a ^7^Li nucleus, which would, in turn, produce in a LiF crystal a track with a length of 4.5 µm (see Figure S2 in Supplementary Material). The probability of these reactions is, however, much lower than that of the (n,α) capture, and again, the tracks of recoiled nuclei might be covered by tracks induced by thermal neutrons (the presence of which may always be expected due to scattering of neutrons). It would be, therefore, advantageous to use in such measurements crystals made of ^7^LiF (i.e., of ^6^Li depleted lithium), insensitive to thermal neutrons, and the growth of such crystals were also undertaken in this work.

## 2. Materials and Methods

### 2.1. Crystal Growth and Detector Preparation

Lithium fluoride, in powder form, as a starting material for crystal growth, was synthesized at IFJ PAN using lithium with different isotopic compositions. Lithium fluoride with a natural isotopic composition was obtained by reacting a saturated solution of lithium chloride (98+% AlfaAesar, Haverhill, MA, USA) with hydrofluoric acid (48% Honeywell/Fluka, Charlotte, NC, USA). The reaction produced a lithium fluoride precipitate, which, after drying, was recrystallized in a muffle furnace at 740 °C. The recrystallization was aimed at converting the fine-crystalline LiF powder with a low bulk density into a powder with a crystal size of 63–212 µm. In turn, enriched isotopes of lithium-6 and lithium-7 were available in metallic form (Li-6) or lithium-7 hydroxide. These raw materials were first converted to lithium chloride and then to lithium fluoride by reaction with hydrofluoric acid. Their preparation was carried out as follows:

Metallic lithium-6 enriched to 95% atoms by weight (Sigma-Aldrich, St. Louis, MO, USA) was used to produce lithium-6 fluoride. The metallic lithium was dissolved with hydrochloric acid to produce lithium chloride. Lithium fluoride was then precipitated with hydrofluoric acid (48% Honeywell/Fluka). The resulting precipitate was recrystallized in a muffle furnace at 740 °C. Lithium-6 fluoride with a grain size of 63–212 µm was used to grow crystals.

Lithium fluoride enriched with lithium-7 was synthesized by starting from lithium-7 hydroxide monohydrate (^7^Li enrichment > 99.92 at. %) (NUKEM Isotopes GmbH, Alzenau, Germany). First, lithium-7 chloride was obtained by a reaction of hydroxide with hydrochloric acid, followed by the production of lithium-7 fluoride through a reaction with hydrofluoric acid. Lithium-7 fluoride was recrystallized in a muffle furnace at 740 °C, and the 63–212 µm grain size fraction was used to grow crystals.

**Figure 3:**
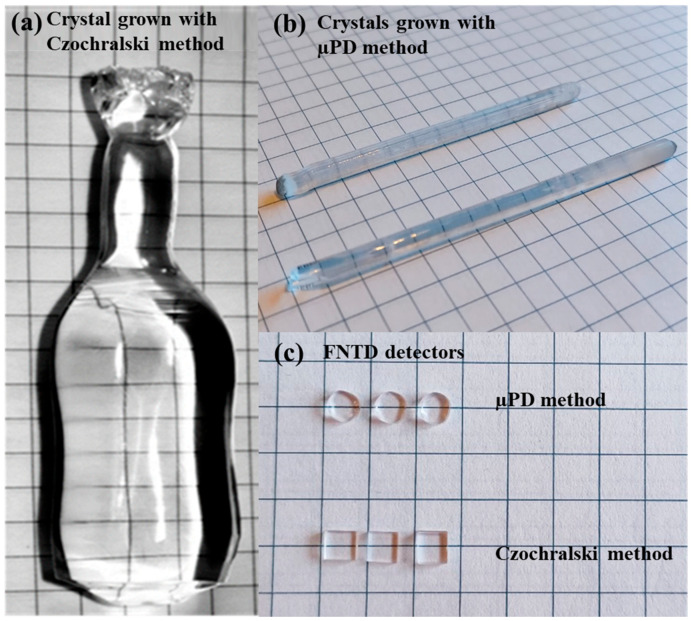
Examples of LiF crystals grown with (**a**) Czochralski method; (**b**) µPD method. Panel (**c**) shows a comparison between FNTD detectors cut from crystals produced with both methods.

LiF crystals were grown at the IFJ PAN (see Figure 3a,b) using a crystal puller made by Cyberstar (Grenoble, France). This equipment enables crystal growth with two techniques: the classic Czochralski method and the micro-pulling-down (µPD) method [12]. Both these techniques were used in the present work. Crystals produced with the µPD method have the form of thin rods with a diameter of up to 3 mm. The growth speed was 0.25 mm/min, and the growth process of a single crystal lasted a few hours. The main advantage of the µPD method is that it requires only a very small amount of the starting material—at the level of a few grams—to obtain a good-quality crystal. This was important as isotopically enriched lithium is a costly material and of limited availability. This applies especially to ^6^Li-enriched material. The disadvantage of this method is that there is no choice of the size of the final sample to be used as a detector—it is determined by the diameter of the grown crystal rod. On the other hand, the Czochralski method enables obtaining much larger crystals, which makes it easier to produce a large number of samples of required dimensions and uniform properties. The disadvantage is that at least 50 g of the starting material was needed and the whole growth process was much longer (several days). With our facility, we were able to produce crystals with diameters up to 3 cm. The typical growth speed was about 3 mm/h and the crystal rotation speed of several to 15 revolutions per minute. In both methods, the growth was performed in the argon atmosphere. It should be emphasized that with both methods, high-quality LiF crystals with similar optical properties were obtained. The choice of method was, therefore, only dictated by the amount of material available and the duration of the growth process.

The obtained crystals were later cut with diamond saws into small samples (see Figure 3c). The standard size of a detector made from a crystal produced with the Czochralski method is about 3 × 3 × 1 mm. The size of the samples cut from crystals grown with the µPD method depends on the diameter of the grown rod. Detectors usually have the form of quasi-circular discs with a diameter of up to 3 mm and a thickness of 1 mm. After cutting, the samples were polished using abrasive straps to obtain perfectly transparent samples and rinsed in acetone in an ultrasonic washer. At the end of the preparation process, all detectors were also annealed for 10 min at a temperature close to the melting point of LiF (840 °C). This process eliminates small scratches originating from polishing and significantly improves the quality of the detector’s surface.

### 2.2. Irradiations of LiF Detectors

Investigations of the response of LiF detectors to neutrons were performed with neutrons from three different energy ranges: thermal (around 0.025 eV), fast (c.a. 1–10 MeV), and high-energy (up to 1000 MeV).

At the IFJ PAN, the neutron test exposures were carried out using a standard radioactive Pu-Be source (emission rate 5 × 10^5^ n.s^−1^). A typical neutron energy spectrum of such a source extends beyond 10 MeV with a maximum between 3 and 5 MeV (see Figure S3a in Supplementary Material) [13]. In order to obtain a thermal neutron field, the source was placed behind a 10 cm layer of polyethylene, which acted as a moderator, slowing down neutrons through their collisions with hydrogen nuclei. The thermal neutron fluence rate was estimated using ^6^LiF:Mg,Ti thermoluminescent detectors, previously calibrated in a reference thermal neutron field [14], and found to be about 4 n.cm^−2^ s^−1^. Some irradiations were performed with the bare Pu-Be source, without any moderator, i.e., with fast neutrons. In that case, the total neutron fluence rate was calculated based on the nominal emission rate and the distance from the source and amounted to 626 n.cm^−2^ s^−1^.

The reference neutron exposures with the neutron doses in terms of personal dose equivalent Hp(10) were performed at the accredited calibration laboratory of the National Centre for Nuclear Research in Otwock using a Pu-Be source (emission rate ~10^7^ n.s^−1^, neutron fluence rate was 128.6 n.cm^−2^ s^−1^) [15]. For irradiations, the detectors were placed on the front surface of a water phantom. This arrangement simulated a dosimeter worn on a human body. The neutrons lose part of their energy through collisions with hydrogen nuclei and some of them are backscattered towards the dosimeter. Such an approach for neutron measurements is known as albedo dosimetry [10].

The exposure to the high-energy neutrons was performed at the CERN-EU high-energy Reference Field (CERF) [16]. The radiation field at CERF is produced by a 120 GeV/c hadron beam hitting a copper target. The resulting neutron energy spectrum is very wide, with the main peak at nearly 100 MeV (see Figure S3b in Supplementary Material) [17].

The detectors were also exposed to the complex cosmic radiation spectrum in space, which consists mainly of high-energy ions of nuclei ranging from hydrogen to iron but also contains a component of secondary neutrons. This was carried out at the Earth orbit onboard the International Space Station in the frame of the DOSIS-3D experiment, as well as at the lunar orbit in the frame of the Artemis-1 mission (MARE experiment). The duration of the exposure was 6 months and 25 days, respectively.

For the studies of temperature effects in LiF crystals, the samples were also irradiated with alpha particles from the Am-241 source (Eckert&Ziegler, Berlin, Germany). Detectors were irradiated using a collimator that ensures a nearly perpendicular angle of incidence. Source activity is around 10.7 MBq, and nominal particle energy is 5.486 MeV. The detectors were irradiated with a fluence of about 1.6 × 10^6^ cm^−2^. 

### 2.3. Microscopic Observations and Image Analysis

Microscopic observations were performed using a Nikon Eclipse Ni wide-field microscope with a CCD DS-Qi2 camera. As a light source, a pE-100 illumination system with 440 nm LEDs (CoolLED), together with a band-pass filter ET445/30, was used. Long-pass filter ET570lp was used for emission light. All images were taken using a 100× TU Plan ELWD (NA 0.80) objective lens. The microscope system is equipped with an adjustable diaphragm that limits the field of view to a quasi-circle with a diameter of 90 µm (observed area of around 6900 µm^2^). 

Almost all images registered after irradiations with neutrons were taken in the form of stacks: a series of pictures obtained with focus set at different depths in the crystal. The first picture in the stack is taken at a given depth, and the next pictures with focus set at an increasingly greater depth in the sample, with a given step. For this study, the first image was usually performed with focus at 6 or 10 µm below the surface, the step was 1 µm, and the thickness of the scanned volume was 20 µm. In this way, for one field of view, the volume of 1.38 × 10^5^ µm^3^ was scanned. The acquisition time for a single image was usually 15 s.

For detectors irradiated with alpha particles, images were registered at the depth of 3 µm under the sample surface. The time of acquisition was 2 s. The intensity of the observed tracks was calculated as the maximum value of the pixel after subtraction of the background signal. The background signal was measured as a modal intensity value in a circle of a radius of 50 pixels (approx. 3.5 μm) around the track. For the studies of the temperature effects, the enhancement factor was introduced, which is a ratio of track intensity after and before applying post-irradiation heat treatment. 

The registered images were then analyzed using ImageJ software, version 1.54f (with Fiji interface) [18]. For the analysis of image stacks, the maximum intensity projections (MIP) were generated, which are a kind of two-dimensional representation of three-dimensional images that enable a fast interpretation of the track geometry. In an MIP, the brightest pixel among all layers of the stack is selected for each pixel position in the stack of images. For numerical background subtraction, a self-developed Fiji plugin was used [19].

## 3. Results and Discussion

### 3.1. Thermal Neutrons

**Figure 4:**
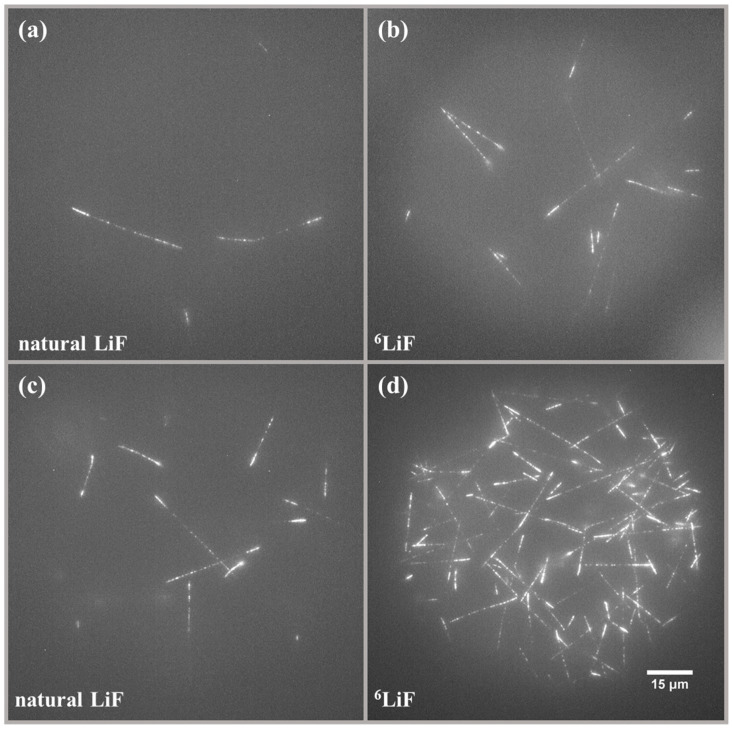
Examples of images registered after irradiations with thermal neutrons. Maximum intensity projection of 21 images taken at depths ranging from 10 µm to 30 µm below the crystal surface for (**a**) natural LiF, neutron fluence c.a. 8.6 × 10^4^ n.cm^−2^; (**b**) LiF enriched with ^6^Li, neutron fluence c.a. 8.6 × 10^4^ n.m^−2^; (**c**) natural LiF, neutron fluence c.a. 4.0 × 10^5^ n.cm^−2^; (**d**) LiF enriched with ^6^Li, neutron fluence c.a. 4.0 × 10^5^ n.cm^−2^.

The previous work demonstrated that the number of visible tracks in LiF crystals increases proportionally with the fluence of thermal neutrons [9]. Figure 4 shows maximum intensity projections of images registered with the detectors made of natural LiF (Figure 4a,c) and LiF enriched with ^6^Li (Figure 4b,d). The tracks visible in the pictures result from a ^6^Li(n,α)^3^H nuclear reaction, as they have a characteristic shape: they consist of two parts, the brighter and shorter part coming from the alpha particle and the longer but less intense part coming from the tritium particle. It is clear that increasing the isotopic content of ^6^Li significantly increases the number of observed tracks and, therefore, decreases the lower detection limit of LiF FNTD detectors.

**Figure 5:**
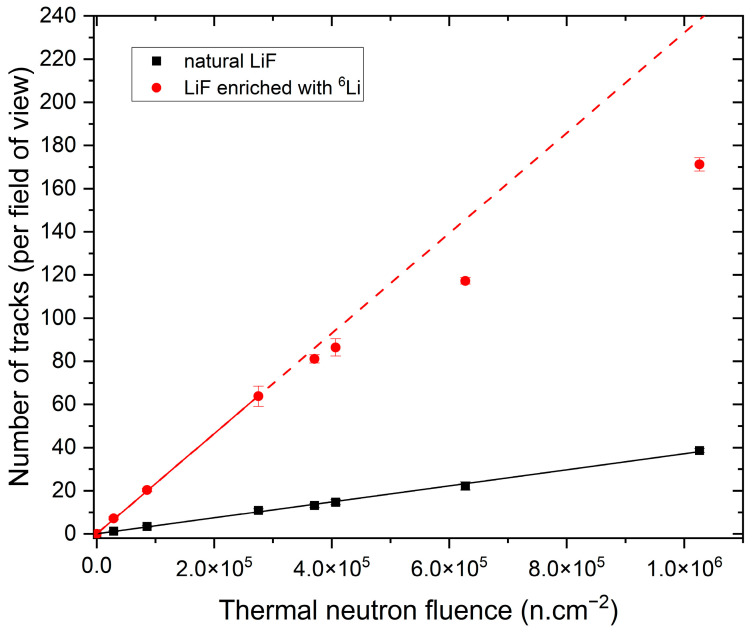
Relationship between a number of the registered tracks per field of view and the thermal neutron fluence for detectors made of natural and ^6^Li-enriched LiF. One track per field of view corresponds to about 7000 tracks per mm^3^.

Figure 5 presents the relationship between the number of registered tracks and the thermal neutron fluence for both types of detectors. For the zero fluence, i.e., the absence of neutrons, no tracks were observed for either natural LiF crystals or ^6^Li-enriched crystals. For the two highest values of neutron fluence, irradiations were also performed using ^7^Li-enriched detectors. However, in that case, practically no tracks were present. This further confirms that the observed tracks are indeed the result of a ^6^Li(n,α)^3^H reaction. As can be seen in Figure 5, the number of registered tracks is about six times higher for crystals enriched with ^6^Li. For this type of detector, the number of tracks increases proportionally with the neutron fluence up to the fluence of about 3.0 × 10^5^ n.cm^−2^. Above this value, the number of tracks present in a single field of view is so high that the tracks overlap, which leads to an underestimation of the counted number of tracks. It seems that for the analysis procedures based on track counting, at this fluence level, the detection limit for ^6^Li-enriched detectors was reached. Since, for the natural LiF crystal, the number of registered tracks is much lower, it is possible to use them for measurements in stronger thermal neutron fields. Assuming that the maximum number of tracks per field of view still possible to count without underestimation is about 70, we can also estimate the maximum measurable fluence for detectors made with natural LiF. Using the linear regression function, we can calculate that the upper detection limit for this type of detector is about 1.9 × 10^6^ n.cm^−2^.

**Figure 6:**
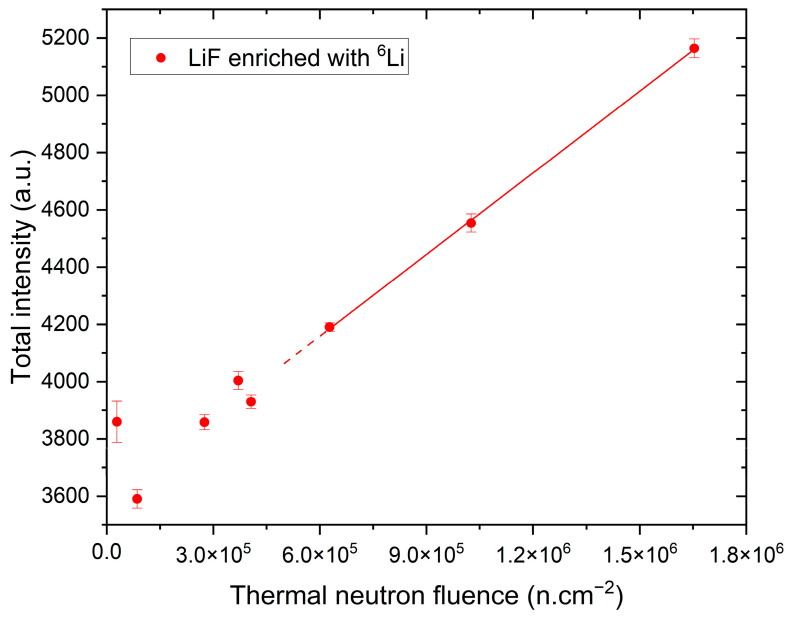
Total fluorescent intensity in the field of view versus thermal neutron fluence for detectors made of LiF enriched with ^6^Li. Calculations were made for the maximum intensity projection of 21 images taken at depths ranging from 10 µm to 30 µm below the crystal surface.

However, track counting is not the only analysis method available for images acquired with FNTDs. Besides that, it is also possible to measure and compare the total intensity of light in a field of view. We checked if this procedure can be used to estimate the dose of thermal neutrons with ^6^LiF FNTDs, especially in neutron fields stronger than 2.8 × 10^5^ n.cm^−2^ (the highest fluence for which the track counting method could be applied without any corrections). Figure 6 shows the relationship between the total intensity in the field of view and the thermal neutron fluence. The linear relationship is present for the fluence values higher than about 5.0 × 10^5^ n.cm^−2^. This indicates that it is indeed possible to use this approach for measurements of high neutron doses. In the case of lower doses and, therefore, a low number of tracks, the total intensity of the photoluminescence signal emitted from tracks cannot be distinguished from the fluctuations of the background noise, and, therefore, this approach is not applicable. In the transition region, i.e., for the fluence values ranging between c.a. 3.0 × 10^5^ n.cm^−2^ and 5.0 × 10^5^ n.cm^−2^, both methods are not linear, and appropriate correction factors should be established. 

**Figure 7:**
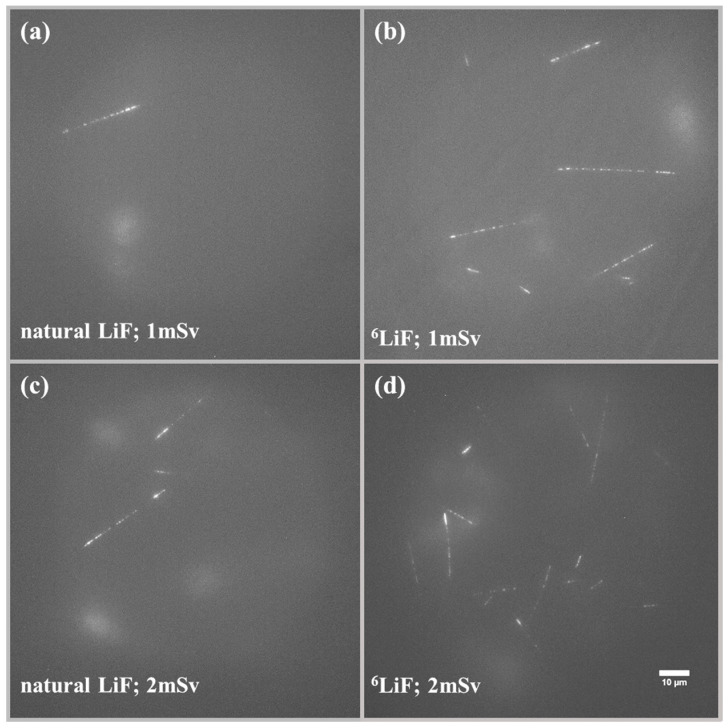
Examples of images registered after irradiations with different individual equivalent doses of neutrons for natural (**a**,**c**) and ^6^Li-enriched (**b**,**d**) detectors. Maximum intensity projection of 21 images taken at depths ranging from 6 to 26 µm under the crystal surface.

**Figure 8:**
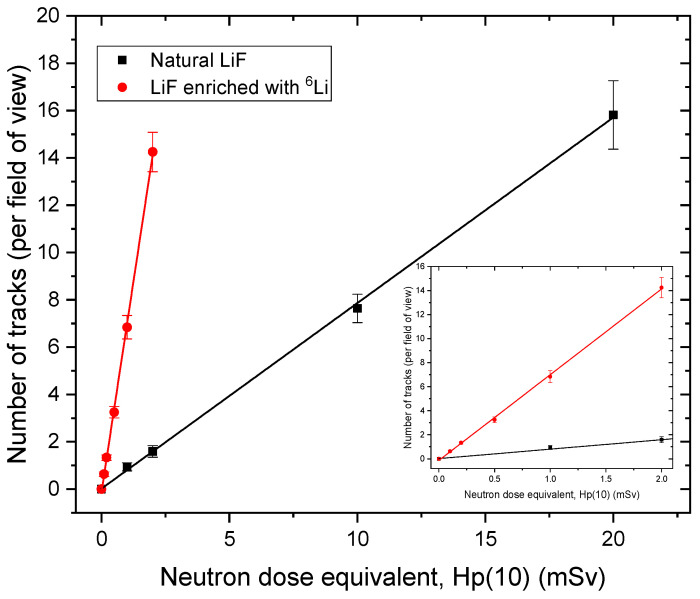
Number of tracks per field of view versus neutron dose equivalent Hp(10) for detectors made of natural and ^6^Li-enriched LiF. The inset graph shows in detail the results in the range up to 2 mSv.

Detectors made of natural LiF and LiF enriched with ^6^Li were also exposed to the reference doses of neutrons in an accredited calibration laboratory. To test their abilities and measurement range for potential applications in personal dosimetry, the crystals were irradiated with individual dose equivalent Hp(10) ranging from 0.1 mSv to 20 mSv. For LiF crystals enriched with ^6^Li, the lower dose range was tested (from 0.1 to 2 mSv), while for natural LiF, the higher (from 1 mSv to 20 mSv). This choice of dose ranges was based on previous experiments performed with these types of detectors. Figure 7 presents differences between typical images registered for different doses with two types of LiF FNTD detectors. Figure 8 shows the dependence of the number of tracks visible in the field of view after irradiation on the individual dose equivalent. It can be seen that enriching the natural LiF with ^6^Li significantly lowers the lower measurement limit. While for natural LiF, reaching the limit of 1 mSv requires scanning a large volume of the detector, for LiF enriched with ^6^Li, it is not necessary, as the higher number of tracks in the field of view provides better statistics while scanning a smaller volume. Thanks to that, even reaching a detection limit as low as 0.1 mSv was possible. Assuming that the maximum number of tracks possible to count without underestimation per field of view is 70, we can estimate that the upper detection limit for detectors made with natural LiF is about 90 mSv and for detectors made of LiF enriched with ^6^Li, about 10 mSv (while using track counting method). To summarize, both types of LiF FNTD detectors have their purposes. ^6^Lienriched crystals are more useful when low doses of neutrons are present as they allow their measurement without the necessity of scanning large volumes, which is time-consuming. However, in strong neutron fields, natural LiF crystals are more practical, as a large number of tracks present in the field of view for ^6^Li-enriched crystals might be hard to analyze due to overlapping and, therefore, lead to underestimation of neutron dose.

### 3.2. Fast Neutrons

**Figure 9:**
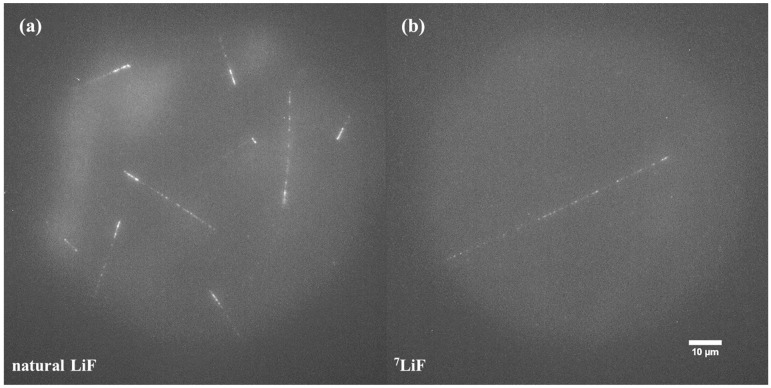
Examples of images registered after irradiations with high energy neutrons at CERF for (**a**) natural LiF crystals and (**b**) ^7^Li-enriched LiF crystals. Maximum intensity projection of 21 images taken at depths ranging from 6 to 26 µm under the crystal surface.

**Figure 10:**
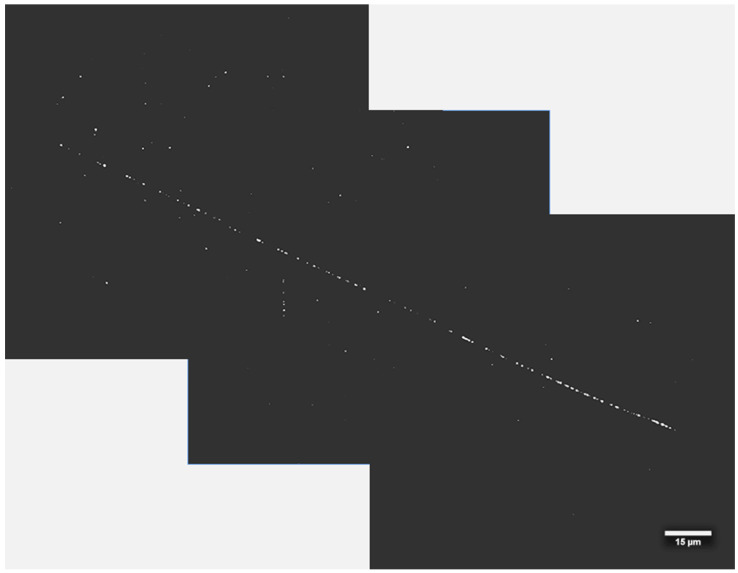
Example of a very long track (about 220 µm) registered with ^7^Li-enriched LiF FNTD detector at CERF. The image consists of a superposition of three maximum intensity projections of image stacks (depth from 11 µm to 15 µm under crystal surface) acquired in the adjacent areas. The image background was numerically subtracted.

Figure 9 shows examples of images acquired for FNTD detectors made from natural (Figure 9a) and ^7^Li-enriched (Figure 9b) LiF crystals, which were irradiated at the CERN-EU high-energy Reference Field (CERF). For detectors made of natural LiF, the number of visible tracks is much higher, and their characteristic shape suggests that the vast majority of tracks result from ^6^Li(n,α)^3^H reaction, meaning that they are caused by low-energy neutrons. A high number of these kind of tracks makes it difficult to observe and register other tracks that may also be present in LiF crystals. On the other hand, for detectors made of ^7^Li-enriched LiF, where ^6^Li is almost not present, the number of tracks is much lower. For the neutron doses used in this study, it often happened that no track was visible in the field of view. The tracks that are present in the images are most probably caused by the recoil nuclei of ^7^Li or even ^19^F if neutron energy is sufficiently high. Those tracks may differ in length depending on the neutron’s energy and scattering angle. For high-energy neutrons such as the ones present at CERF (peak energy 100 MeV), the tracks coming from lithium nuclei can have a length of even several hundreds of µm (see Figure S2 in Supplementary Material). Figure 10 shows the track registered for ^7^Li-enriched LiF crystal. The presented picture consists of three separate microscopic images shifted relative to each other by 60 µm in the horizontal plane and 30 µm in the vertical plane. The total length of the track, also considering its angle to the crystal’s surface, is about 220 µm. This range in LiF corresponds to a ^7^Li nucleus with an energy of about 45 MeV, which might be knocked out by a neutron with an energy of about 100 MeV or higher. As illustrated in Figure S3b (Supplementary Material), such neutrons are present in the CERF spectrum. 

**Figure 11:**
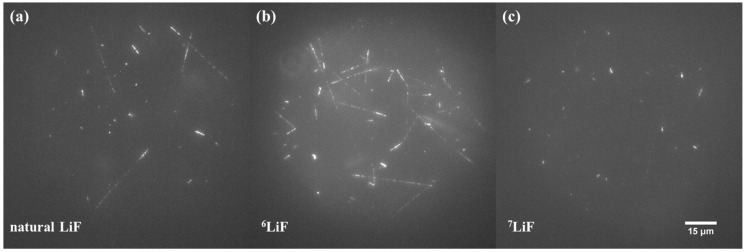
Comparison of images acquired after irradiation with fast neutrons from Pu-Be source for (**a**) natural LiF, (**b**) LiF enriched with ^6^Li, and (**c**) LiF enriched with ^7^Li. Maximum intensity projection of 21 images taken at depths ranging from 10 to 30 µm under the crystal surface.

The tracks of recoil nuclei in LiF may also be observed for neutrons with lower energies than those in the CERF. Figure 11 shows examples of images registered with LiF crystals with different isotopic compositions after irradiations with fast neutrons from the Pu-Be source. For natural LiF (Figure 11a), it is possible to distinguish two types of tracks: long tracks with characteristic shapes coming from thermal neutrons, which are present due to neutron scattering, and shorter tracks of various lengths coming from recoil nuclei after a collision of a fast neutron with a Li nucleus. As in previously conducted experiments, the number of tracks is highest for ^6^Li-enriched LiF crystals due to the higher probability of reactions with thermal neutrons. Shorter tracks coming from recoil nuclei are also present, but they are hard to see and analyze as they are often covered by longer tracks (see Figure 11b). Using ^7^Li-enriched crystals enables easier analysis of tracks resulting from recoil nuclei. Tracks resulting from reactions with thermal neutrons are not present due to the absence of ^6^Li, which makes images clearer and other tracks more apparent (see Figure 11c). 

### 3.3. Cosmic Radiation

**Figure 12:**
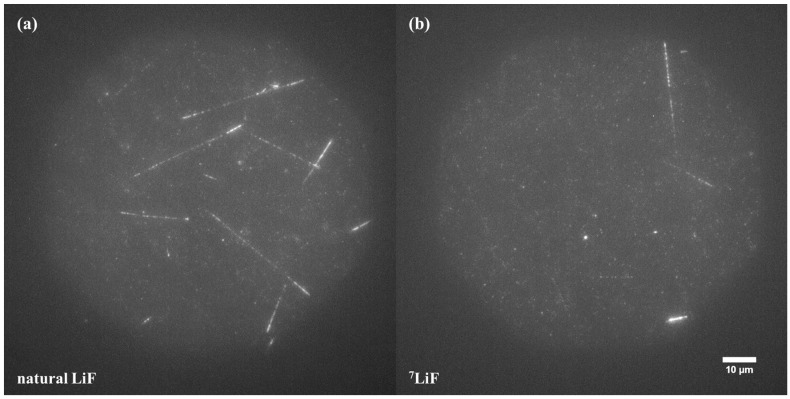
Comparison of images obtained after 6-month exposure of FNTDs on the International Space Station in Earth orbit: (**a**) natural LiF and (**b**) LiF enriched with ^7^Li. The pictures show maximum intensity projection of 21 images taken at depths ranging from the crystal surface to 21 µm under the surface. Acquisition time for a single image was 20 s.

**Figure 13:**
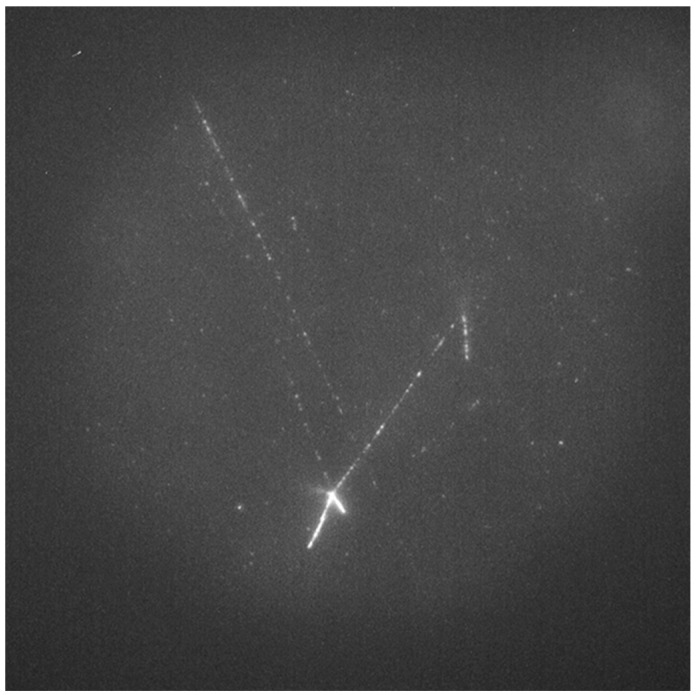
Example of an image for ^7^Li-enriched LiF crystal after exposure during the flight to the lunar orbit in the frame of the MARE experiment (Artemis-1 mission). Maximum intensity projection of 21 images taken at depths ranging from 6 to 26 µm under the crystal surface. The acquisition time for a single image was 30 s.

Similarly to the high-energy neutron fields, also in space radiation measurements, it may be beneficial to use ^7^Li-enriched LiF crystals to simplify image analysis by removing the high number of tracks resulting from reactions with low-energy neutrons. Figure 12 shows a comparison between the images obtained for natural and ^7^Li enhanced LiF crystals, which spent 6 months on the International Space Station in Earth orbit. In Figure 12a, which shows the image for natural LiF, tracks coming from ^6^Li(n,α)^3^H reactions are numerous and bright. For that reason, they may overlap and cover other tracks, mostly created by ions. For ^7^Li-enriched crystals (Figure 12b), analysis is easier, as ion-induced tracks are easier to distinguish in the absence of tracks resulting from low-energy neutrons. Figure 13 shows another example of cosmic radiation tracks: the image registered for a ^7^LiF crystal exposed on the lunar orbit in the frame of the MARE experiment. One can see tracks in the shape of several lines, some of them very bright, converging at one point. This pattern suggests a nuclear reaction (nucleus fragmentation) due to a collision with an ultra-high-energy particle of the cosmic radiation spectrum. 

### 3.4. Temperature Effects

**Figure 14:**
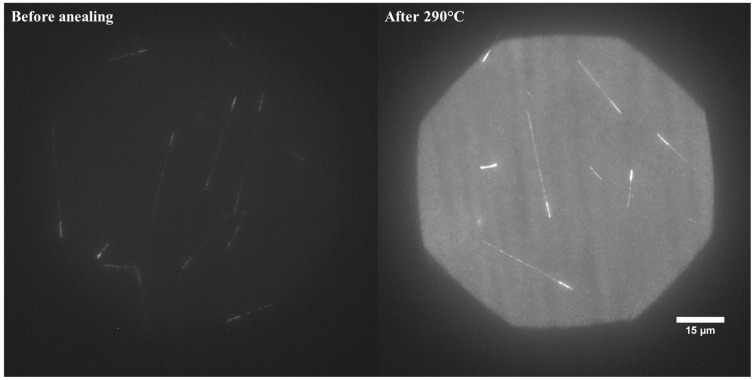
Influence of post-irradiation annealing at 290 °C on track intensity. Maximum intensity projection for stacks of images acquired at depths ranging from the surface to 20 µm in 1 µm steps for the same LiF crystal before and after heating at 290 °C. The acquisition time for a single image 5 s. The sample was irradiated with thermal neutrons (moderated Pu-Be source). The brightness, contrast, and other graphic parameters of both images are the same.

For all LiF crystals, regardless of their isotopic composition, annealing at 450 °C for 30 min leads to complete removal of the photoluminescence signal [20]. However, it was previously found that a heat treatment at a lower temperature may, in certain conditions, lead to signal enhancement [21]. The mechanism leading to this effect is still unclear. In our previous studies, we demonstrated that post-irradiation annealing at 290 °C for 3 min can cause a mean track intensity improvement by a factor of 2.5. This can be especially useful for neutron detection purposes, as the tritium tracks, which are a product of ^6^Li(n,α)^3^H reaction, have low intensity and may sometimes be hard to distinguish from the background. Figure 14 illustrates the impact of the amplification effect on tracks registered with LiF crystals after irradiations with thermal neutrons. Although the background also increases after post-irradiation annealing, the contrast between tracks and background is much better, which makes image analysis easier.

All of the studies previously published were performed for natural LiF crystals produced with the Czochralski method. To test if the isotopic composition and crystal growth method has an impact on the occurrence of the enhancement effect and its strength, a series of measurements was conducted. Two detectors were chosen from each of the following types: natural LiF crystals made with the Czochralski method, natural LiF crystals made with the µPD method, ^6^Li-enriched LiF crystals made with the µPD method, ^7^Li-enriched LiF crystals made with the Czochralski method, and ^7^Li-enriched LiF crystals made with the µPD method. All detectors were irradiated with alpha particles from the Am-241 source. After irradiations, microscopic observations were performed. For each crystal, at least five images, taken at a depth of 3 µm under the surface, were taken, and mean track intensity was calculated. Then, the detectors were submitted to the post-irradiation annealing using the Linkam THMS600 heating stage. Crystals were placed at room temperature on the heating stage and heated to the temperature of 290 °C with a heating rate of 150 °C/min. After reaching that temperature samples were annealed for 3 min. Then, the heating was turned off. Samples were cooled, still on the heating stage, for another 3 min. After that, they were removed from the heating stage and placed on the aluminum plate to further cool down. After annealing, the microscopic observations were repeated, and the enhancement factor was calculated. For all detectors, regardless of their isotopic composition or growth method, the mean track intensity before post-irradiation annealing was similar and was within ±10% of the average value (see Table 1). Small differences like that are normal even for detectors made of the same crystal and are usually a result of statistical errors, non-ideal background subtraction, or differences in a cutting and polishing process. This means that the chosen crystal growth method does not affect the sensitivity of produced FNTD detectors. Table 1 shows the enhancement factors obtained for different types of LiF FNTD detectors.

The enhancement factors for natural LiF and ^7^Li-enriched detectors are similar and range from 2.47 to 3.07. Slightly higher results obtained for detectors enriched with ^7^Li are rather an incidental result of individual differences that are present for different crystals and even for different samples made from the same crystal (see enhancement factors for two different natural LiF crystals made with the Czochralski method). It is, however, worth noting that the highest enhancement factors (c.a. 3.0) were observed for crystals made with the µPD method. Somewhat in contrast to these results are the results that were measured for the ^6^LiF crystals, for which the enhancement factor was found to be significantly lower, not exceeding 1.9, despite the crystals being manufactured using the µPD method. This smaller amplification effect should probably not be attributed to the different isotopic composition. A more likely explanation might be the presence of impurities, which could be introduced into the material during the process of isotopic separation.

## 4. Conclusions

In this work, we described the properties of LiF crystals grown using Li of different isotopic compositions from the standpoint of their application as fluorescent nuclear track detectors used in measurements in the neutron radiation fields.

In the case of thermal neutrons, ^6^LiF detectors registered about six times more tracks of (n,α) reaction products than natural LiF. The number of tracks increases linearly with increasing thermal neutron fluence for both these detectors. For ^6^LiF, the upper limit of linearity is about 3.0 × 10^5^ cm^−2^. For higher fluences tracks overlap, which leads to underestimation of their number. For natural LiF, the upper limit was estimated to be 1.9 × 10^6^ cm^−2^. It was found that for a very high number of tracks, it is possible to measure the neutron fluence using the total intensity of the emitted light instead of track counting.

In the case of fast neutrons, the tracks of recoiled lithium and fluorine nuclei can be successfully employed for measurement purposes. For this purpose, ^7^LiF detectors have been demonstrated to be particularly effective, as the absence of ^6^Li nuclei allows for the avoidance of tracks induced by thermal neutrons, which are always present even in the nominal fast neutron spectrum. This also applies to the measurement of cosmic radiation in space, where tracks created by energetic ions may be overlapped by tracks due to thermal neutrons if natural LiF is used, and ^7^LiF detectors are again the optimal solution.

Summarizing, all three studied isotopic compositions of LiF have their specific areas of application in radiation measurements with FNTD methods:

- Natural LiF detectors are the best solution for applications in strong thermal neutron fields (e.g., dosimetry at nuclear reactors or experimental fusion facilities). They might also be a cost-effective solution for all general applications if a very high sensitivity to thermal neutrons is not required;
- ^6^Li detectors are the best choice for measurements of very low doses of thermal neutrons (applications in personal dosimetry);
- ^7^Li allows the analysis of tracks of different particles, as well as high-energy neutrons, even in the presence of a strong thermal neutron field (cosmic radiation, accelerators).

Furthermore, aside from the response to neutron radiation, no other significant differences in the crystal properties were identified, irrespective of the isotopic composition and crystal growth method employed.

## Figures and Tables

**Table 1:** Enhancement factors calculated for different samples of LiF crystals irradiated with alpha particles.

Isotopic Composition	Growth Method	Series Number of the Crystal Sample	Mean Track Intensity Before Post-Irradiation Annealing (a.u.)	Enhancement Factor
Natural LiF	Czochralski method	590_2018_1	1374	2.69
		684_2019_1	1413	2.47
	µPD method	531_2018_1	1402	2.99
		531_2018_2	1421	3.04
^7^Li-enriched LiF	Czochralski method	790_2021_1	1592	2.79
		790_2021_2	1605	2.72
	µPD method	534_2018_1	1461	2.89
		536_2018_1	1367	3.07
^6^Li-enriched LiF	µPD method	660_2019_1	1644	1.65
		660_2019_2	1622	1.82

## Data Availability

Data will be made available on request.

## Supplementary Materials

The following supporting information can be downloaded at https://www.mdpi.com/article/10.3390/ma17205029/s1, Figure S1: Microscopic cross sections of ^6^Li and ^7^Li for neutron absorption; Figure S2: Maximum range in LiF crystal of ^7^Li and ^19^F nuclei recoils after collisions with neutrons vs. neutron energy calculated with the SRIM computer code; Figure S3: (a) Typical neutron energy spectrum for Pu-Be source; (b) Neutron energy spectrum of CERN-EU high-energy Reference Field (CERF). Refs. [22,23] can be found in Supplementary Materials.
