# Supplementary material for: Isotopically Enriched Lithium Fluoride Crystals for Detection of Neutrons with the Fluorescent Track Technique
Source: Materials (Basel). 2024 Oct 14;17(20):5029. doi: 10.3390/ma17205029 (PMC11509241; doi:10.3390/ma17205029)
Supplement: Supplementary file 1 [file materials-17-05029-s001.zip › materials-3250593-supplementary.pdf]

Supplementary material

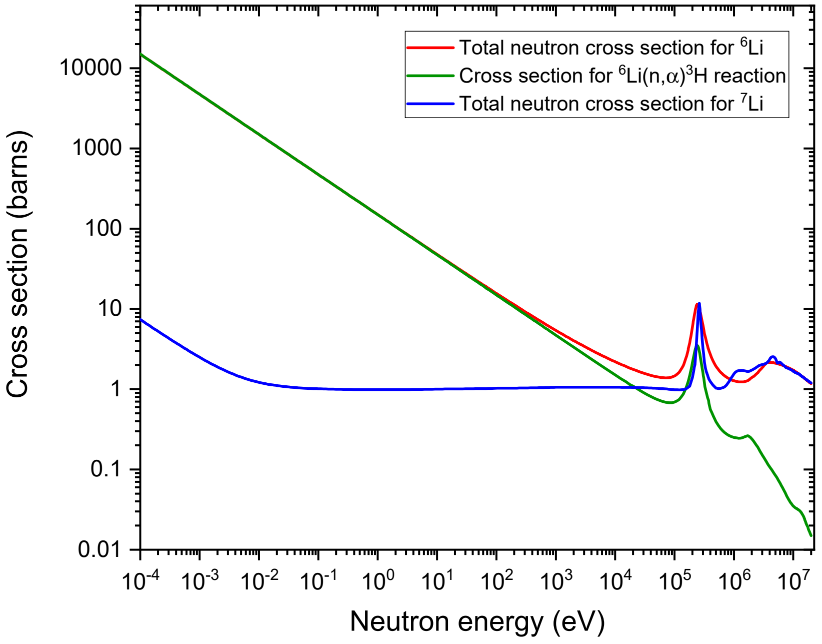

Figure S1. Microscopic cross sections of ^{6}Li and ^{7}Li for neutron absorption.[1]

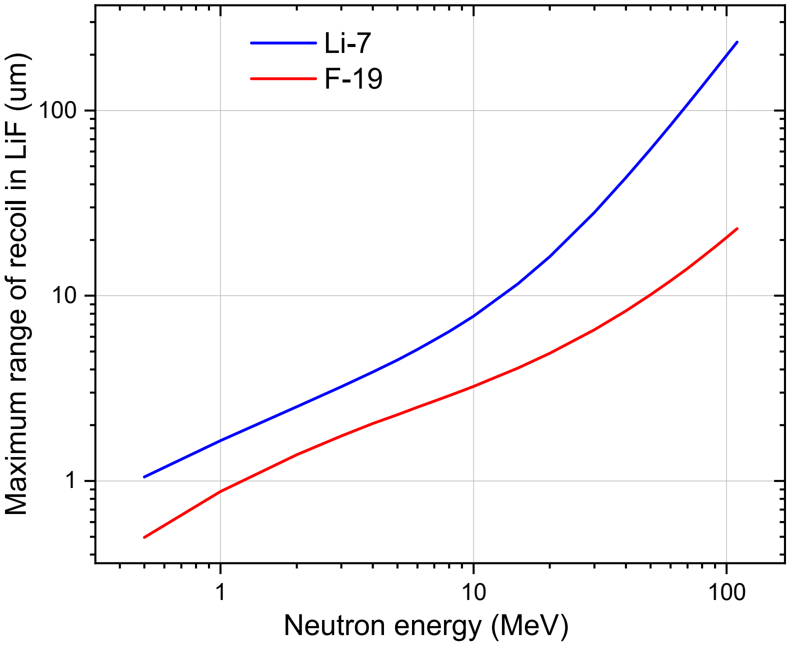

Figure S2. Maximum range in LiF crystal of ^{7}Li and ^{19}F nuclei recoils after collisions with neutrons vs. neutron energy calculated with the SRIM [2] computer code.

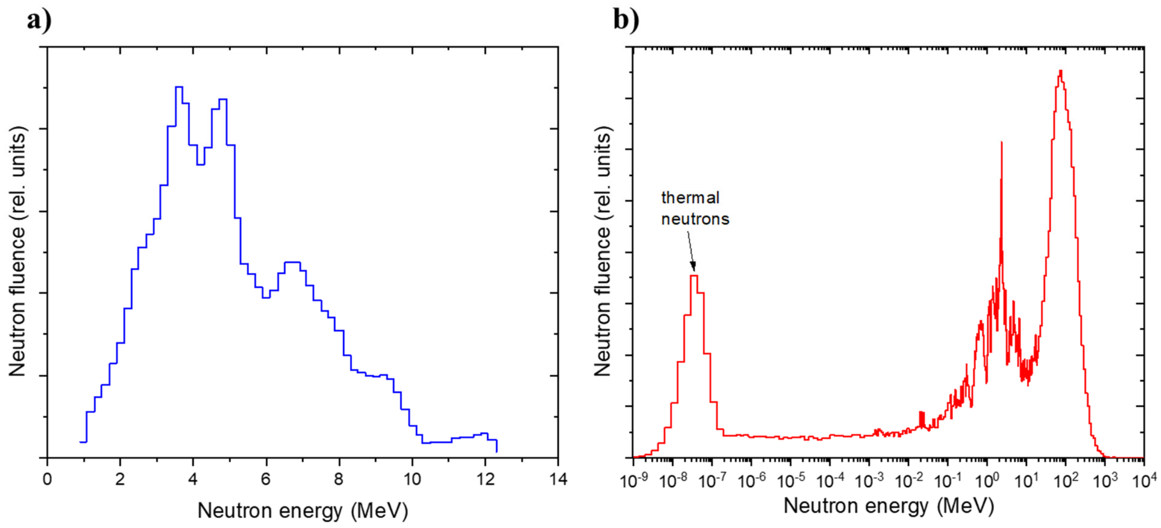

Figure S3. a) Typical neutron energy spectrum for Pu-Be source [3]; b) Neutron energy spectrum of CERN-EU high-energy Reference Field (CERF) [4].

References

1. Brown, D. A.; Chadwick, M. B.; Capote, R.; Kahler, A. C.; Trkov, A.; Herman, M. W.; Sonzogni, A. A.; Danon, Y.; Carlson, A. D.; Dunn, M.; Smith, D. L.; Hale, G. M.; Arbanas, G.; Arcilla, R.; Bates, C. R.; Beck, B.; Becker, B.; Brown, F.; Casperson, R. J.; Conlin, J.; Cullen, D. E.; Descalle, M. A.; Firestone, R.; Gaines, T.; Guber, K. H.; Hawari, A. I.; Holmes, J.; Johnson, T. D.; Kawano, T.; Kiedrowski, B. C.; Koning, A. J.; Kopecky, S.; Leal, L.; Lestone, J. P.; Lubitz, C.; MĂˇrquez DamiĂˇn, J. I.; Mattoon, C. M.; McCutchan, E. A.; Mughabghab, S.; Navratil, P.; Neudecker, D.; Nobre, G. P. A.; Noguere, G.; Paris, M.; Pigni, M. T.; Plompen, A. J.; Pritychenko, B.; Pronyaev, V. G.; Roubtsov, D.; Rochman, D.; Romano, P.; Schillebeeckx, P.; Simakov, S.; Sin, M.; Sirakov, I.; Sleaford, B.; Sobes, V.; Soukhovitskii, E. S.; Stetcu, I.; Talou, P.; Thompson, I.; van der Marck, S.; Welser-Sherrill, L.; Wiarda, D.; White, M.; Wormald, J. L.; Wright, R. Q.; Zerkle, M.; Ĺ˝erovnik, G.; Zhu, Y., ENDF/B-VIII.0: The 8th Major Release of the Nuclear Reaction Data Library with CIELO-project Cross Sections, New Standards and Thermal Scattering Data. *Nuclear Data Sheets* **2018,** 148, 1-142.
2. Ziegler J F, Z. M. D.; Biersack, J. P., SRIM–the stopping and range of ions in matter. *Nucl. Instrum. Methods. B* **2010,** 268, 1818.
3. Söderström, P. A.; Matei, C.; Capponi, L.; Açıksöz, E.; Balabanski, D. L.; Mitu, I. O., Characterization of a plutonium–beryllium neutron source. *Appl. Radiat. Isot.* **2021,** 167, 109441.
4. Van Hoey, O.; De Saint-Hubert, M.; Parisi, A.; Caballero-Pacheco, M. Á.; Domingo, C.; Pozzi, F.; Froeschl, R.; Stolarczyk, L.; Olko, P., Evaluation and modelling of the lithium fluoride based thermoluminescent detector response at the CERN-EU high-energy reference field (CERF). *Radiat. Meas.* **2023,** 162, 106923.
